# Generation of angiostatin-like fragments from plasminogen by prostate-specific antigen

**DOI:** 10.1038/sj.bjc.6692167

**Published:** 1999-12

**Authors:** H-H Heidtmann, D M Nettelbeck, A Mingels, R Jäger, H-G Welker, R E Kontermann

**Affiliations:** St Joseph Hospital, Wiener Strasse 1, Bremerhaven D-27568, Germany; Institut für Molekularbiologie und Tumorforschung andZentrum für Innere Medizin, Abt. Hämatologie und Onkologie, Philipps-Universität Marburg, Baldingerstraße, Marburg D-35033, Germany; Biochemisches Institut, Klinikum der Justus Liebig Universität Gießen, Friedrichstr. 24, Gießen D-35385, Germany

**Keywords:** angiostatin, PSA, plasminogen, prostate cancer, anti-angiogenesis

## Abstract

Angiostatin, a potent inhibitor of angiogenesis, tumour growth and metastasis, is a biologically active fragment of plasminogen, containing the kringle domains 1–4. It is generated from plasminogen by limited proteolysis. We show that prostate-specific antigen (PSA), a serine proteinase secreted by human prostate and human prostate cancer cells, is able to convert Lys-plasminogen to biologically active angiostatin-like fragments, containing kringles 1–4, by limited proteolysis of peptide bond Glu439–Ala440 in vitro. In an in vitro morphogenesis assay, the purified angiostatin-like fragments inhibited proliferation and tubular formation of human umbilical vein endothelial cells with the same efficacy as angiostatin. This finding might help to understand growth characteristics of prostate cancer, which usually has low microvessel density and slow proliferation. © 1999 Cancer Research Campaign


*British Journal of Cancer* (1999) **81**(8), 1269–1273
© 1999 Cancer Research Campaign
Article no. bjoc.1999.0840

### Generation of angiostatin-like fragments from plasminogen by prostate-specific antigen

**H-H Heidtmann[1], DM Nettelbeck[3], A Mingels[3], R Jäger[3], H-G Welker[4] and RE Kontermann[2]**

[1]St Joseph Hospital, Wiener Strasse 1, D-27568 Bremerhaven, Germany; [2]Institut für Molekularbiologie und Tumorforschung and [3]Zentrum für Innere Medizin, Abt. Hämatologie und Onkologie, Philipps-Universität Marburg, Baldingerstraße, D-35033 Marburg, Germany; [4]Biochemisches Institut, Klinikum der Justus Liebig Universität Gießen, Friedrichstr. 24, D-35385 Gießen, Germany

**Summary** Angiostatin, a potent inhibitor of angiogenesis, tumour growth and metastasis, is a biologically active fragment of plasminogen, containing the kringle domains 1–4. It is generated from plasminogen by limited proteolysis. We show that prostate-specific antigen (PSA), a serine proteinase secreted by human prostate and human prostate cancer cells, is able to convert Lys-plasminogen to biologically active angiostatin-like fragments, containing kringles 1–4, by limited proteolysis of peptide bond Glu439–Ala440 in vitro. In an in vitro morphogenesis assay, the purified angiostatin-like fragments inhibited proliferation and tubular formation of human umbilical vein endothelial cells with the same efficacy as angiostatin. This finding might help to understand growth characteristics of prostate cancer, which usually has low microvessel density and slow proliferation. © 1999 Cancer Research Campaign

**Keywords:** angiostatin; PSA; plasminogen; prostate cancer; anti-angiogenesis

Angiostatin is an inhibitor of angiogenesis, a process which is necessary for tumour growth and metastasis. It consists of the first four of five kringle domains of plasminogen (O'Reilly et al, 1994) and can be generated by the proteolytic cleavage of plasminogen by metalloelastase (matrix metalloproteinase 12) (Dong et al, 1997). Matrix metalloproteinases 3, 7, and 9 have been reported also to produce angiostatin-like peptides from plasminogen (Patterson and Sang, 1997; Lijnen et al, 1998). Furthermore, in the media of prostate cancer cell lines, angiostatin-generating activity has been found (Gately et al, 1996). However, this activity was not attributed to PSA. Rather, plasminogen activators in the obligatory presence of free sulphydryl donors, were shown to convert plasminogen to angiostatin, requiring the activation of plasminogen (Gately et al, 1997).

Prostate-specific antigen (PSA) is a kallikrein-like serine proteinase. Under physiological conditions, it is secreted extracorporally into seminal fluid, its target substrate being semenogelin. Three cleavage sites have previously been identified between amino acid residues Tyr44–Thr45, Leu84–His85, and Tyr136–Ser137 in the secreted semenogelin I (Lilja et al, 1989). A more detailed analysis identified a total of 12 cleavage sites in semenogelin I and seven sites in semenogelin II (Malm et al, 1997).

Only minor amounts of PSA reach the bloodstream from the prostate and are inhibited by the plasma proteinase inhibitors α-2-macroglobulin (Leinonen et al, 1996) and α-1-antichymotrypsin (Lilja et al, 1991). Whereas most tumour-associated proteinases (like cathepsins B and D, matrix metalloproteinases, plasminogen activators etc.) are overexpressed enzymes of ubiquitous origin, the extraordinary specificity of PSA as a tumour proteinase derives from the fact that no human cell types secrete significant amounts of PSA other than prostatic glandular cells or prostate cancer cells.

We now show that PSA converts Lys-plasminogen to biologically active angiostatin-like fragments by cleavage of a single peptide bond between kringle domains 4 and 5.

*Received 25 September 1998*
*Revised 17 May 1999*
*Accepted 8 June 1999*

*Correspondence to:* H-H Heidtmann

### MATERIALS AND METHODS

### Materials

PSA was purified from seminal plasma according to Zhang et al (1995). Affinity absorption on Trasylol-Sepharose (Pharmacia) was added to the procedure according to Christensson et al (1990) in order to remove possible traces of kallikrein-like proteinases other than PSA from the preparation. Activity was monitored with MeO–Suc–Arg–Pro–Tyr–NA (S-2586, Chromogenix). PSA was electrophoretically pure and > 90% active. Identity and purity of PSA were confirmed by N-terminal amino acid sequencing. Electrophoretically pure human Lys-Plasminogen (Plasminogen HS, Activity >90%. Charge No. 281189) was kindly provided by Dr Römisch from the research department of Behringwerke (Marburg, Germany).

### Proteolytic cleavage of plasminogen

To determine proteolysis of plasminogen by PSA, 3 μg of PSA were incubated with 50 μg plasminogen in 10 mM Tris–HCl, pH 8.0, in a final volume of 10 μl at 37°C. For time course analysis, samples were started individually at different times so as to reach their planned end points simultaneously, in order to avoid artefacts. At end point, all samples were immediately taken up in reducing sodium dodecyl sulphate polyacrylamide gel electrophoresis (SDS-PAGE) sample buffer, boiled for 2 min and electrophoresed on 10% polyacrylamide gels (Laemmli, 1970).

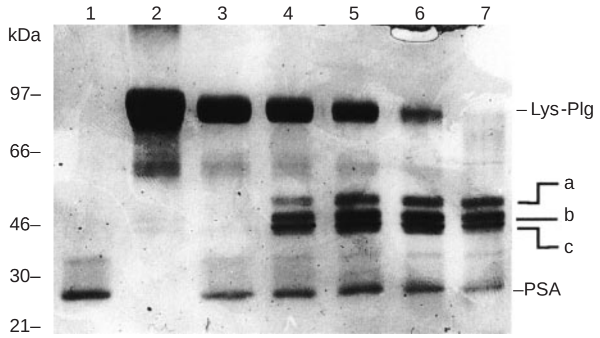

**Figure 1** Time course of plasminogen degradation by PSA, 37°C, pH 8.0. Lane 1: PSA only, 3 µg, at 6 h. Lane 2: plasminogen only, 50 µg, at 6 h. Lanes 3 to 7: plasminogen with PSA, at 0 h, 1 h, 2 h, 4 h, 6 h. Ten per cent reducing SDS-PAGE

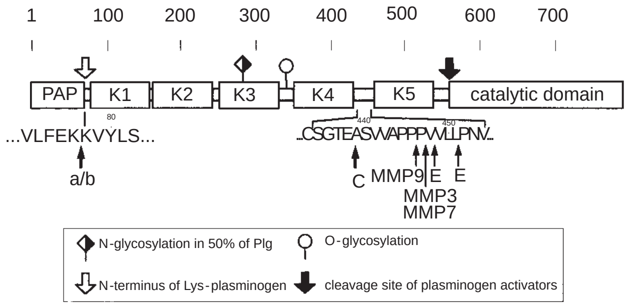

**Figure 3** Plasminogen domain structure showing the N-termini of fragments a, b, and c. PAP: Preactivation peptide. K1–5: Kringle domains 1–5. The cleavage sites of metalloproteinases MMP-3, -7 and -9 as well as pancreatic elastase (E) are indicated

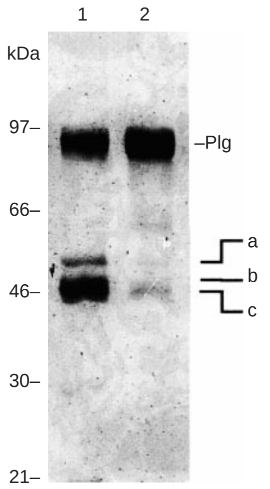

**Figure 2** Comparison of active versus inactivated PSA. Lane 1: 3 µg of PSA incubated with 50 µg of plasminogen in 10 mM Tris–HCl, pH 8.0, at 37°C for 6 h. Ten per cent reducing SDS-PAGE. Lane 2: same protocol with PSA heat-inactivated (100°C for 30 min)

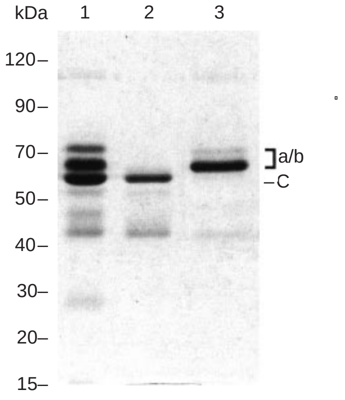

**Figure 4** Purification of angiostatin-like fragments. Plasminogen digested with PSA to completion (lane 1) was purified by lysine sepharose chromatography. Fragment c was found in the flow-through (lane 2), while fragments a and b corresponding to the angiostatin-like fragments bound to the resin and were eluted in the presence of 200 mM ε-aminocaproic acid (lane 3). Fragments were analysed by 10% non-reducing SDS-PAGE

Controls consisting of plasminogen and PSA alone were treated like the test samples. They showed no change after 6 h of incubation. For control purposes, PSA was inactivated by either 48-h exposure to 50 mM DFP (Sigma) or by boiling for 30 min. The inactivated preparations showed no detectable activity on MeO–Suc–Arg–Pro–Tyr–NA even after prolonged incubation (24 h).

### N-terminal protein sequencing

For N-terminal amino acid sequencing of fragments, 30 µg of PSA were mixed with 500 µg of plasminogen in a final volume of 90 µl of 10 mM Tris–HCl, pH 8.0. After incubation at 37°C for 6 h, 90 µl of reducing SDS-sample buffer was added and the sample was boiled for 2 min. Finally, the material was applied to seven wells of a 10% PAGE gel, electrophoresed and blotted to a polyvinyldithioride (PVDF) membrane. After brief staining with Coomassie blue, the membrane was destained in 50% methanol, bands were cut out and pooled. The N-termini of the fragments were determined by automated Edman degradation using the Protein Sequencer 477A (Applied Biosystems).

### Purification of angiostatin-like fragments

Angiostatin-like fragments were purified by lysine sepharose chromatography (Shi and Wu, 1988). Three milligrams of plasminogen were digested with PSA for 20 h to completion as described above. The sample was then loaded onto a lysine sepharose column (Pharmacia) equilibrated with 10 mM Tris–HCl pH 8.0, and the flow-through was collected. Bound proteins were eluted with 200 mM ε-aminocaproic acid. Peak fractions were dialysed against phosphate-buffered saline (PBS), and the protein concentrations were determined photometrically. For controls, angiostatin was generated by elastase digestion of plasminogen according to O'Reilly et al (1996).

### In vitro morphogenesis assay

Human umbilical vein endothelial cells (HUVEC) were purchased from Cell-Systems (Clonetics). The cells were cultured in endothelial basal medium (EBM) supplemented with 2% brain extract (Clonetics). All experiments were performed with cells from passages 2–6, incubated at least overnight in EBM in absence

 *© 1999 Cancer Research Campaign*

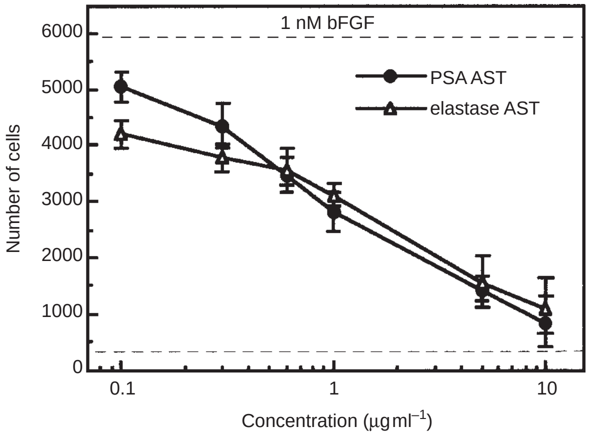

**Figure 5** In vitro morphogenesis assay. Inhibition of endothelial cell proliferation analysed after tubular formation: affinity-purified fragments a and b (PSA AST); elastase-generated angiostatin (elastase AST)

of growth factor-containing brain extract. Matrigel (100 ml) diluted 1:2.5 with EBM containing 1 nM basic fibroblast growth factor (bFGF) (R&D) was added to each well of 24-culture plates at 4°C and then incubated at 37°C for 30 min to induce gelation. HUVEC ($5 \times 10^4$) were then plated into each well in EBM with and without different stimulants. The cells were then incubated for 18 h at 37°C. Formation of tubes was evaluated first by phase contrast microscopy and second by cell counting after treatment of cells with dispase (Kumar et al, 1998).

### RESULTS

Incubation of Lys-plasminogen with PSA generated a set of three distinct fragments by limited proteolysis at 37°C. SDS-PAGE analysis of the time course of this reaction is shown in Figure 1. The three major bands (a, b, and c), with apparent molecular masses of 44.5 kDa, 41 kDa and 38 kDa, appeared already after the first hour. With time, the intensity of these bands increased, while the band corresponding to plasminogen was diminished simultaneously. After prolonged incubation over 6 h, the fragments a, b, and c persisted without further degradation, whereas plasminogen was consumed almost completely. No cleavage of plasminogen was observed when active PSA was substituted by heat-inactivated PSA (Figure 2) or PSA inactivated with DFP (not shown).

In order to identify the cleavage sites, fragment bands were blotted onto PVDF membrane, cut out and analysed for their N-terminal amino acid sequences. As summarized in Figure 3, both fragments a and b had the same N-termini (KVYLSEKK) starting with Lys78. A minor fraction within both fragments started with Val79, due to a common heterogeneity at this site (Wallén and Wiman, 1972). Sequencing of fragment c (ASVVAPPPVV) indicated a cleavage site at Glu439–Ala440, located between kringles 4 and 5. Sequencing of the bands corresponding to Lys-plasminogen and PSA confirmed their identities.

Angiostatin-like fragments a and b were purified by lysine sepharose chromatography. Fragment c was found in the flow-through, while fragments a and b bound to the resin and could be eluted with ε-aminocaproic acid (Figure 4). This finding further demonstrated that fragments a and b contain the kringle-like domains. In in vitro morphogenesis assays, fragments a and b inhibited tubular formation and proliferation of HUVECs with the same efficacy as elastase-generated angiostatin (Figure 5). Half-maximal inhibition in this assay was observed at a concentration of 1 µg ml$^{-1}$. Morphologic assessment by phase-contrast microscopy showed full inhibition of tubular formation (Figure 6).

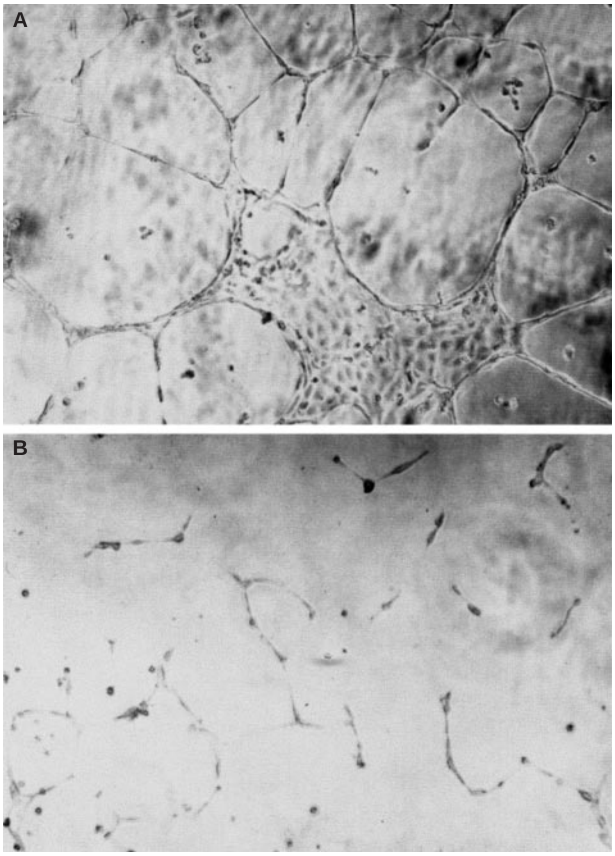

**Figure 6** In vitro morhogenesis assay. Inhibition of tubular formation as analysed by phase contrast microscopy. (**A**) Medium containing 1 nM bFGF. (**B**) Medium containing 1 nM bFGF and 10 µg ml$^{-1}$ affinity purified fragments a and b

### DISCUSSION

Both fragments a and b had the same N-terminal amino acid sequence, which corresponds to the N-terminus of angiostatin and was identical to that of the starting material, Lys-plasminogen. The molecular masses of fragments a and b, as determined by SDS-electrophoresis, were 44.5 kDa and 41 kDa respectively. This is large enough to cover the kringle domains 1–4 (approximately 370 amino acids), of which angiostatin consists (Figure 3). Since fragment c resulted from a unique cleavage of peptide bond Glu439–Ala440, which is located between kringles 4 and 5, it is most likely that both fragments a and b have identical amino acid backbones spanning from Lys78 to Glu439, for which a calculated molecular mass of 41.2 kDa would be expected. The difference in molecular mass between fragments a and b is attributable to a variable glycosylation of plasminogen at Asn289: only 50% of plasminogen molecules are glycosylated at this site with 10–11 monosaccharide units (Hayes and Castellino, 1979).

The molecular mass of fragment c was determined by SDS-electrophoresis as 38 kDa. This corresponds to a calculated molecular

© 1999 Cancer Research Campaign mass of 38.5 kDa for the remainder of the plasminogen molecule, spanning from Ala440 to the C-terminal Asn791, comprising kringle 5 and the catalytic domain and resembling mini-plasminogen (Sottrup-Jensen et al, 1978).

We concluded that both fragments a and b correspond to angiostatin and are generated from Lys-plasminogen by cleavage of a single peptide bond between kringles 4 and 5 by PSA. The affinity-purified fragments a and b had angiostatin-like activity as shown by the inhibition of tubular formation and proliferation of HUVECs in the in vitro morphogenesis assays.

Fragment c, the remaining part of plasminogen, was not cleaved at Arg561, indicating that activation of plasminogen to plasmin had not taken place. This distinguishes the action of PSA on plasminogen from the mechanism described by Gately et al (1996), who showed that human prostate carcinoma cell lines PC-3, DU-145 and LNCaP express enzymatic activity that can generate bioactive angiostatin from plasminogen. They did not attribute this activity to PSA, however, which is only secreted in significant amounts from LNCaP (Hasenson et al, 1989). Rather, they identified urokinase released by PC-3 cells to be sufficient for angiostatin generation by autocatalysis of plasmin – however, only in the presence of free sulphydryl donors (Gately et al, 1997). Thus, it seems that some prostate cancer cells may have two different means of angiostatin generation: urokinase, which requires strong reducing agents for artificial disruption of intramolecular disulphide-bridges and the activation of plasmin, and PSA, which does not. Considering the specificity of PSA for the extracorporal catalysis of semenogelin, there being no known physiological intracorporal substrate for PSA, dystopic release of PSA in prostate cancer could theoretically lead to paraneoplastic formation of angiostatin-like activity. Thus, we interpret our finding not as a physiological but rather an erroneous, pathophysiological process, which imitates angiostatin generation by the ubiquitous macrophage proteinases.

Different enzymes are capable of conversion of plasminogen to angiostatin. Dong et al (1997) showed a role for macrophage metalloelastase (MME) in the generation of angiostatin in Lewis lung carcinoma. In vitro, matrix metalloproteinase MMP-9 cleaves between kringles 4 and 5 at peptide bond Pro446–Pro447 (Patterson and Sang, 1997), and MMP3 (Lijnen et al, 1998) as well as MMP-7 (Patterson and Sang, 1997) both cleave at the neighbouring peptide bond, Pro447–Val448. This is 7–8 amino acids downstream from the cleavage site of PSA observed by us. Pancreatic elastase cleavage sites were mapped to Val449–Val450 and Leu451–Pro452 (Cao et al, 1997). Specificity of the PSA-cleavage site in our experiments was confirmed by the fact that it was completely distinct from any other known proteinase cleavage site in this domain.

Apparently, the scissile peptide chain between kringles 4 and 5 is exposed to the surface of the plasminogen molecule, allowing for proteolytic attack by proteinases of different specificities and classes. This resembles the bait region in α-2-macroglobulin (Sottrup-Jensen et al, 1981). The cleavage site of PSA in this area is novel and has not been described for any other enzyme. Since several forms of angiostatin-like plasminogen fragments with microheterogeneity in this scissile connection peptide region between kringles 4 and 5 are generated by different proteinases, angiostatin may be no longer considered a single entity. Rather, there are multiple isoforms depending on the mechanism of generation.

We showed for the first time that PSA purified from human seminal plasma specifically interacts with plasminogen to form angiostatin-like fragments from plasminogen in a defined, cell-free system. However, the physiological role of this finding remains elusive. Is PSA released by prostate cancers likely to get in contact with large plasma proteins like plasminogen? Alpha-2-macroglobulin is the major inhibitor of PSA when it reaches the bloodstream (Leinonen et al, 1996). Some PSA in the plasma of prostate cancer patients is complexed with α-2-macroglobulin, requiring that at least some PSA released from prostate cancer enters the bloodstream in active form. Therefore, PSA may theoretically react with plasminogen before being inhibited by plasma proteinase inhibitors. Further, in areas of invasive cancer, plasma extravasation may take place, with molecules like plasminogen (92 kDa) diffusing more readily than the very large α-2-macroglobulin (800 kDa). Local concentrations of PSA in tumour tissue may be sufficiently high to cause proteolysis of plasminogen. Even after prolonged incubation, the fragments from limited PSA proteolysis were not degraded any further.

In the future, it will be interesting to determine whether generation of angiostatin by PSA actually does occur in vivo. Since angiostatin can induce and sustain dormancy of tumours (O'Reilly et al, 1996), our findings might help to understand the general clinical observation that prostate cancer has a very low progression rate.

### ACKNOWLEDGEMENTS

We are grateful for the very skilled technical assistance of Margitta Alt, and for helpful discussion with Professor Rudolf Egbring and Dr Weingärtner.

### REFERENCES

Cao Y, Chen A, An SSA, Ji RW, Davidson D, Cao Y and Llinás M (1997) Kringle 5 of plasminogen is a novel inhibitor of endothelial cell growth. *J Biol Chem* **272**: 22924–22928

Christensson A, Laurell C and Lilja H (1990) Enzymatic activity of prostate-specific antigen and its reactions with extracellular serine proteinase inhibitors. *Eur J Biochem* **194**: 755–763

Dong Z, Kumar R, Yang X and Fidler IJ (1997) Macrophage-derived metalloelastase is responsible for the generation of angiostatin in Lewis lung carcinoma. *Cell* **88**: 801–810

Gately S, Twardowski P, Stack MS, Patrick M, Boggio L, Cundiff DL, Schnaper HW, Madison L, Volpert O, Bouck N, Enghild J, Kwaan HC and Soff GA (1996) Human prostate carcinoma cells express enzymatic activity that converts human plasminogen to the angiogenesis inhibitor, angiostatin. *Cancer Res* **56**: 4887–4890

Gately S, Twardowski P, Stack MS, Cundiff DL, Grella D, Castellino FJ, Enghild J, Kwaan HC, Lee F, Kramer RA, Volpert O, Bouck N and Soff GA (1997) The mechanism of cancer-mediated conversion of plasminogen to the angiogenesis inhibitor angiostatin. *Proc Natl Acad Sci USA* **94**: 10868–10872

Hasenson M, Lundh B, Stege R, Carlstrom K and Pousette A (1989) PAP and PSA in prostatic carcinoma cell lines and aspiration biopsies: relation to hormone sensitivity and to cytological grading. *Prostate* **14**: 83–90

Hayes ML and Castellino FJ (1979) Carbohydrate of the human plasminogen variants *J Biol Chem* **254**: 8772

Kumar R, Yoneda J, Bucana CD and Fidler IJ (1998) Regulation of distinct steps of angiogenesis by different angiogenic molecules. *Int J Oncol* **12**: 749–757

Laemmli UK: Cleavage of structural proteins during the assembly of the head of bacteriophage T4 (1970) *Nature* (Lond.) **227**: 680–685

Leinonen J, Zhang WM and Stenman UH (1996) Complex formation between PSA isoenzymes and protease inhibitors. *J Urol* **155**: 1099–1103

Lilja HR, Abrahamsson P-A and Lundwall A (1989) Semenogelin, the predominant protein in human semen. *J Biol Chem* **264**: 18894–1900

 © 1999 Cancer Research Campaign

Lilja HR, Christensson A, Dahlen U, Matikainen MT, Nilsson O, Pettersson K and Lövgren T (1991) Prostate specific antigen in human serum occurs predominantly in complex with alpha-1-antichymotrypsin. *Clin Chem* **37**: 1618–1625

Lijnen HR, Uguwu F, Bini A and Collen D (1998) Generation of an angiostatin-like fragment from plasminogen by stromelysin-1 (MMP-3). *Biochemistry* **37**: 4699–4702

Malm J, Hellman J and Lilja H (1997) Mapping of the unique enzyme characteristics of PSA. *J Urol* **157**: 345

O'Reilly MS, Holmgren L, Shing Y, Chen C, Rosenthal RA, Moses M, Lane WS, Cao Y, Sage EH and Folkman J (1994) Angiostatin: a novel angiogenesis inhibitor that mediates the suppression of metastases by a Lewis lung carcinoma. *Cell* **79**: 315–328

O'Reilly MS, Holmgren L, Chen C and Folkman J (1996) Angiostatin induces and sustains dormnacy of human primary tumours in mice. *Nat Med* **2**: 689–692

Patterson BC and Sang QA (1997) Angiostatin-converting enzyme activities of human matrilysin (MMP-7) and gelatinase B/type IV collagenase (MMP-9). *J Biol Chem* **272**: 28823–28825

Shi GY and Wu HL (1988) Isolation and characterization of microplasminogen. A low molecular weight form of plasminogen. *J Biol Chem* **263**: 17071–17075

Sottrup-Jensen L, Claeys H, Zajdel M, Petersson TE and Magnusson S (1978) The primary structure of human plasminogen: isolation of two lysine-binding fragments and one 'mini'-plasminogen (MW 38 000) by elastase-catalyzed-specific limited proteolysis. In: *Progress in Chemical Fibrinolysis and Thrombolysis*, Vol. 3, Davidson JF, Rowan RM, Samama MM and Desnoyers PC (eds), pp. 191. Raven Press: New York

Sottrup-Jensen L, Lonblad PB, Stepanik TM, Petersen TE, Magnusson S and Jornvall H (1981) Primary structure of the 'bait' region for proteinases in alpha 2-macroglobulin. Nature of the complex. *FEBS Lett* **127**: 167–173

Wallén P and Wiman B (1972) Characterization of human plasminogen. II. Separation and partial characterization of different molecular forms of human plasminogen. *Biochem Biophys Acta* **257**: 122–129

Zhang WM, Leinonen J, Kalkkinen N, Dowell B and Stenman UH (1995) Purification and characterization of different forms of prostate-specific antigen in human seminal fluid. *Clin Chem* **41**: 1567–1573

*© 1999 Cancer Research Campaign*
